# A convolutional neural network for high throughput screening of femoral stem taper corrosion

**DOI:** 10.1177/09544119231177834

**Published:** 2023-06-09

**Authors:** Anastasia M Codirenzi, Brent A Lanting, Matthew G Teeter

**Affiliations:** 1 School of Biomedical Engineering, Western University, London, ON, Canada; 2 Division of Orthopaedic Surgery, Department of Surgery, Schulich School of Medicine & Dentistry, Western University, London, ON, Canada; 3 Department of Medical Biophysics, Schulich School of Medicine & Dentistry, Western University, London, ON, Canada

**Keywords:** Machine learning, arthroplasty, trunnionosis, corrosion

## Abstract

Corrosion at the modular head-neck taper interface of total and hemiarthroplasty hip implants (trunnionosis) is a cause of implant failure and clinical concern. The Goldberg corrosion scoring method is considered the gold standard for observing trunnionosis, but it is labor-intensive to perform. This limits the quantity of implants retrieval studies typically analyze. Machine learning, particularly convolutional neural networks, have been used in various medical imaging applications and corrosion detection applications to help reduce repetitive and tedious image identification tasks. 725 retrieved modular femoral stem arthroplasty devices had their trunnion imaged in four positions and scored by an observer. A convolutional neural network was designed and trained from scratch using the images. There were four classes, each representing one of the established Goldberg corrosion classes. The composition of the classes were as follows: class 1 (*n* = 1228), class 2 (*n* = 1225), class 3 (*n* = 335), and class 4 (*n* = 102). The convolutional neural network utilized a single convolutional layer and RGB coloring. The convolutional neural network was able to distinguish no and mild corrosion (classes 1 and 2) from moderate and severe corrosion (classes 3 and 4) with an accuracy of 98.32%, a class 1 and 2 sensitivity of 0.9881, a class 3 and 4 sensitivity of 0.9556 and an area under the curve of 0.9740. This convolutional neural network may be used as a screening tool to identify retrieved modular hip arthroplasty device trunnions for further study and the presence of moderate and severe corrosion with high reliability, reducing the burden on skilled observers.

## Introduction

Trunnionosis refers to the fretting and corrosion of modular hip arthroplasty devices at the head-neck taper junction. This process can release debris that generates adverse tissue reactions and can ultimately cause failure of the hip arthroplasty.^1–4^ Trunnionosis is believed to be underreported, and is usually described from implant retrieval studies.^1^ The presence of corrosion and fretting on explanted hip arthroplasty devices can be quantified using the Goldberg scoring method.^5^ Implant retrieval studies have helped identify areas for advancement in implant design, manufacturing, and installation, and they are of importance to identify specific device issues that may have been previously unknown.^3,4,6–17^ Large-scale retrieved studies in knee arthroplasty have given new insight to drivers of wear, but similarly large studies for hip arthroplasty yet to be completed.^12^

The Goldberg scoring method requires a trained observer to classify the corrosion and/or fretting class on the head and the taper typically under low-power microscopy. This method is time consuming and prohibits large scale study of retrieved arthroplasty devices. Studies that have looked at trunnionosis generally observe less than 150 implants, and often less than 100.^3,4,6–8,14,15,18^ Centers that do not have a research space must ship their implants to centers that do. This is logistically intensive and leads to infrequent collaboration between centers and limitations in the ability of centers to participate in implant orthopedic research without an already established program.

Automation of corrosion detection would allow centers to collect data on explanted implants and identify implants that may be suitable for further analysis. It would reduce the labor-intensive aspect of Goldberg scoring and allow for more careful use of shipping implants and logistics. Machine learning has been successfully used in several imaging and corrosion detection classifications, and this may be extended to use for wear detection on medical devices.^19–24^ Milimonfared et al. described an automated corrosion scoring approach with 85% accuracy, however, their method required substantial image pre-processing, and was trained using few trunnions of a specific design.^24^

The purpose of the present investigation is to create a novel machine learning pipeline using a convolutional neural network applied to images of the femoral stem taper that can rapidly identify implants with moderate to severe corrosion that could be selected for further investigation.

## Methods

### Implant imaging and visual scoring

**Figure 1.**
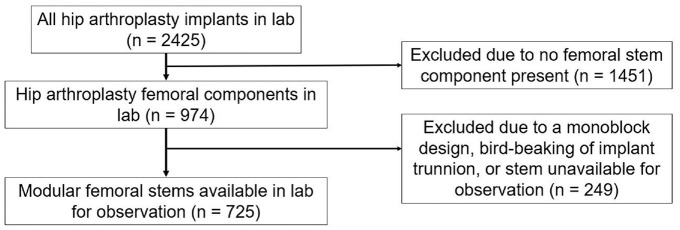
Study design and included implants.

All hip arthroplasty implants in our institutional implant retrieval laboratory were reviewed for inclusion (Figure 1). Implants included for imaging were designs with a modular head-neck taper where the femoral stem was retrieved at the time of revision surgery. Excluded were implants that were non-modular, and cases that had gross taper failure (“bird-beaking”). Each stem was imaged using via digital microscope at 20x magnification (DSX1000, Olympus Cooperation, Tokyo, Japan) at 1200 × 1200 and in RGB color. The taper surface was divided into four areas (medial, lateral, posterior, and anterior) with each area represented by one image. Images were taken with the aid of an image diffuser when possible, as this minimized the amount of metallic glare. Imaging of the implants was completed by the team engineer and no scoring was done during the imaging and there was a cooling off period of a month between the images being taken and the subsequent scoring.

**Figure 2.**
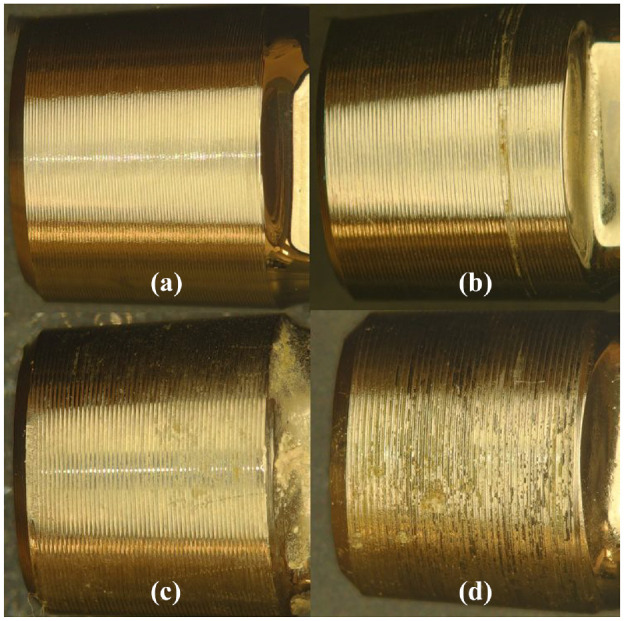
Digital microscopy images (20x magnification, DSX1000, Olympus Cooperation, Tokyo, Japan) at 1200 × 1200 and in RGB color that represent each class Representative images of each class (a) class 1 (N=no visible corrosion observed), (b) class 2 (<30% of taper surface discolored or dull), (c) class 3 (>30% of surface discolored or dull, or,<10% of taper surface containing black debris, pits, or etch marks), (d) class 4 (>10% of taper surface containing black debris, pits, or etch marks).

The microscopy images (Figure 2) were then examined and assigned a corrosion score using the method of Goldberg et al., described in Table 1. Only the corrosion level according to the Goldberg scale was determined. The more specific type of corrosion requires advanced imaging (i.e. Scanning Electron Microscopy) to be conducted.^25^ This was not undertaken due to the magnitude of implants studied in this project and the subsequent labor and testing cost associated with doing so. A single score was assigned to each image and recorded. Images were scored independently of other images in the trunnion set. The scoring was done by an engineer. Images were excluded if they were not of sufficient quality (unfocused, glare that obstructed view of 30% or more of the surface). A subset of 100 images from the test set was provided to a secondary observer, a fellowship trained arthroplasty surgeon, to check for reliability of scoring between observers and calculated using the interclass correlation coefficient (ICC) with a 95% confidence level. For both scorers, the images were viewed at the size and with the observer in control of the length of time the image was observed. Scoring was done independently with no sharing of scoring between the observers until later ICC analysis.

**Table 1.** Goldberg scoring criteria.^5^

	Class 1	Class 2	Class 3	Class 4
Severity	None	Mild	Moderate	Severe
Criteria	No visible corrosionobserved	<30% of taper surfacediscolored or dull	>30% of surfacediscolored or dull, or, <10%of taper surface containing blackdebris, pits, or etch marks	>10% of taper surfacecontaining black debris,pits, or etch marks

### Data curation

Images were sorted into classes based on their Goldberg score. Ten percent of the full dataset was separated out to create a testing set, keeping the proportion to their representation within the class. In line with the best practice guidelines for machine learning for medical devices, this testing set was maintained separately from the training/validation set.^26^ The data was organized into three datasets each with a training/validation set and an associated test set (Table 2). The same images were included in each of the separate datasets but with the images reorganized into different class groupings. This was done to best understand the appropriate use case for the network, understand how the network handled the different groupings, and the limitations of the network. The testing set was also organized into the associated class groupings as they corresponded to the training sets used. In all cases the training/validation set and the testing set were kept separate.

**Table 2.** Description of the different datasets.

Dataset	Dataset description
1. Each corrosion class (class 1 vs 2vs 3vs 4)	All images included in their respective Goldberg corrosion class(class 1, class 2, class 3, class 4)
2. No corrosion versus corrosion (class 1 vs classes 2, 3, and 4)	All images included, separated into two classes, no corrosion andcorrosion. No corrosion is comprised of class 1 images and corrosionis comprised of class 2, 3, and 4 images.
3. None to mild corrosion versus moderate to severe corrosion (classes 1 and 2 vs classes 3 and 4)	No/mild corrosion class comprised of class 1 and 2 images andmoderate/severe corrosion class comprised of class 3 and 4 images.

### Neural network architecture

**Figure 3.**
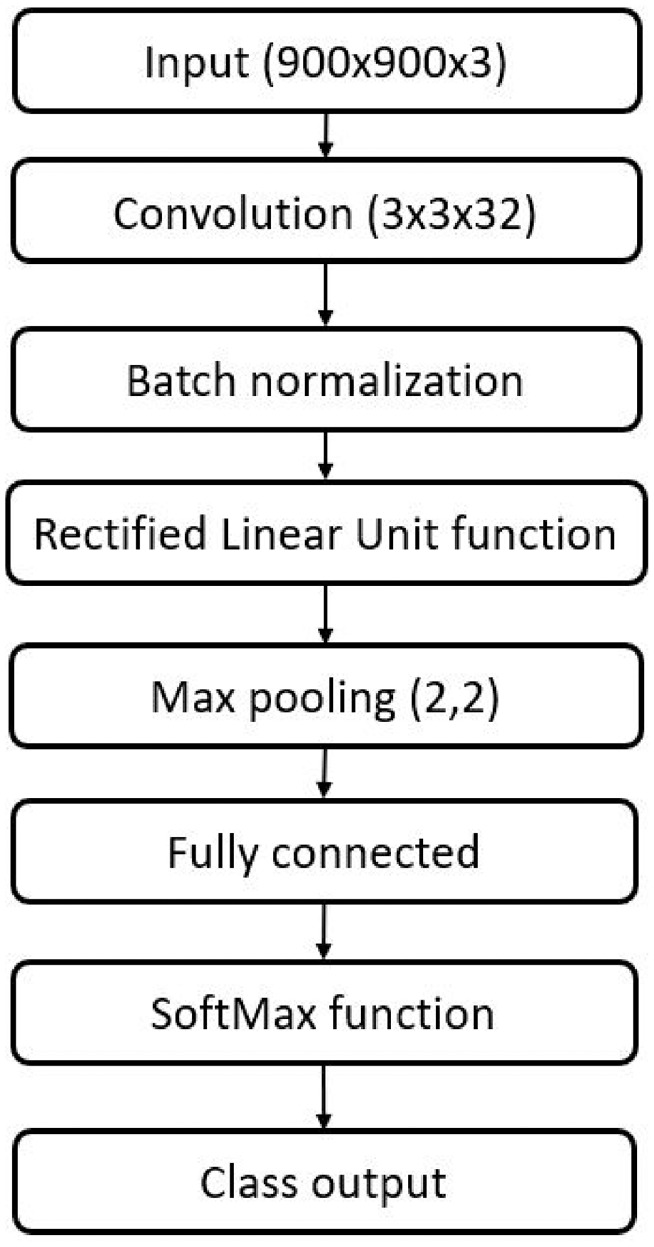
Convolutional neural network architecture. The neural network is comprised of an input of 900 ’ 900 images with RGB coloring. It then has a convolutional layer with a filter size of 3 × 3 × 32. Batch normalization was then employed. Then had a rectified linear unit activation function. That led to a max pooling layer with a filter size of 2 × 2 and a stride of 1,1. Then it had the fully connected layer, SoftMax function, and the output layer.

A convolutional neural network was designed and trained from scratch using MATLAB’s DeepNetwork Designer application (MATLAB for Windows, version 2021b). The network architecture was based off the concept of starting with a small network and expanding outward using a trial-and-error method, first using extreme cases (i.e. Class 1 versus Class 4) and then including intermediate cases. A network diagram for the network used in this study is shown in Figure 3. It utilizes a single convolutional layer for feature learning. A single convolutional layer was maintained due to the image size (900 × 900 px) which was the smallest size possible while retaining sufficient detail for annotation by observers. The same network architecture and training parameters were used for all datasets.

Three regularization techniques were used: batch normalization, L2 regularization, and early-stopping. Batch normalization normalizes along a mini-batch of the data across all observations for each color channel independently. Batch normalization was applied after the first convolutional layer and before the activation function to allow normalization of the output before the fully connected layer.^27,28^ L2 regularization works by adding a term to the error function which prevents overfitting. Early stopping refers to ending the training before the determined number of epochs to prevent overfitting and generally an early stop is used when stagnation in the loss is observed during training.^29^ Variation in the inclusion of early stopping, L2 regularization, and the batch normalization are included in the Supplemental Information section.

### Network training and testing

The same network architecture was trained separately using each of the three curated datasets. As previously described, 10% of all images were split to create the holdout testing set. The training/validation set had 15% randomly split to create the validation set, with the remainder being used as the training set. The convolutional neural network was trained separately using each dataset using the version with all images. For the training parameters, an ADAM optimizer was used with a learning rate of 0.003. All networks were given 14 epochs to train and the images were shuffled every epoch. The images were read in with a mini-batch size of 15 images and validation was done every 25 iterations. An L2 regularization of 0.001 was used. Additional variations of the optimizer and learning rate are available in the Supplemental Information section.

Each trained network was tested using both versions of its associated test dataset, one with all images and one with images only taken with an image diffuser. Accuracy and sensitivity were computed. Accuracy refers to the overall proportion of the images that were correctly classified. Sensitivity refers to the proportion of images the network classified correctly for each class.^29^ Confusion matrices were used to show the classifications made in each class, both correctly and incorrectly. The reliability of the neural network was evaluated by plotting the receiver operating characteristic curve (ROC) and determining the area under the curve (AUC). An area under the curve of 0.7–0.8 is considered acceptable, 0.8–0.9 is considered excellent, and more than 0.9 is considered outstanding.^30^

## Results

### Imaging and corrosion scoring

In total, 725 stems were microscopically imaged in four positions for a total of *n* = 2890 images, with *n* = 10 excluded due to poor image quality. The images were assigned a Goldberg corrosion score of class 1 (*n* = 1228), class 2 (*n* = 1225), class 3 (*n* = 335), and class 4 (*n* = 102). The interclass correlation coefficient for interobserver scoring was calculated to be 0.60 (±0.13), rating as moderately reliable. The test set comprised *n* = 298 images, with *n* = 2592 remaining in the training/validation set. Table 3 summarizes the content of each dataset, where the classifier was used to separate images into different classes.

**Table 3.** Distribution of the images within each datasets.

Dataset	All images		Images taken with an image diffuser
	Training/Validation	Testing	Testing
1	Class 1 (*n* = 1100)Class 2 (*n* = 1101)Class 3 (*n* = 301)Class 4 (*n* = 90)	Class 1 (*n* = 128)Class 2 (*n* = 125)Class 3 (*n* = 34)Class 4 (*n* = 12)	Class 1 (*n* = 93)Class 2 (*n* = 95)Class 3 (*n* = 27)Class 4 (*n* = 9)
2	Class 1 (*n* = 1100)Class 2, 3, 4 (*n* = 1492)	Class 1 (*n* = 128)Class 2, 3, 4 (*n* = 170)	Class 1 (*n* = 93)Class 2, 3, 4 (*n* = 131)
3	Class 1, 2 (*n* = 2201)Class 3,4 (*n* = 391)	Class 1, 2 (*n* = 252)Class 3,4 (*n* = 46)	Class 1,2 (*n* = 188)Class 3,4 (*n* = 36)

### Neural network training and evaluation

**Figure 4.**
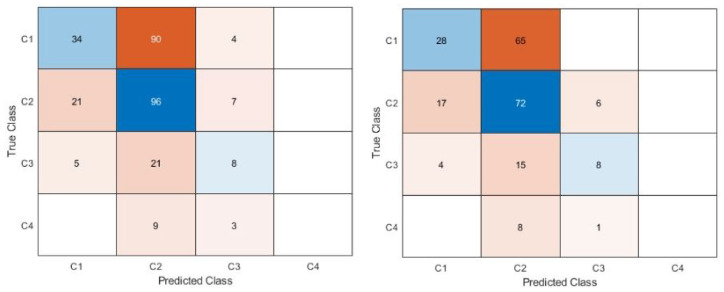
Confusion matrix for dataset 1 (class 1 vs class 2 vs class 3 vs class 4). Left is all images, right is with glare removed. The blue diagonal shows correct classifications (predicted class matches the true class) while the orange off-diagonal shows misclassifications. Intensity of color is based off count in each category. The proportion of misclassifications is higher for the model that included glare.

**Figure 5.**
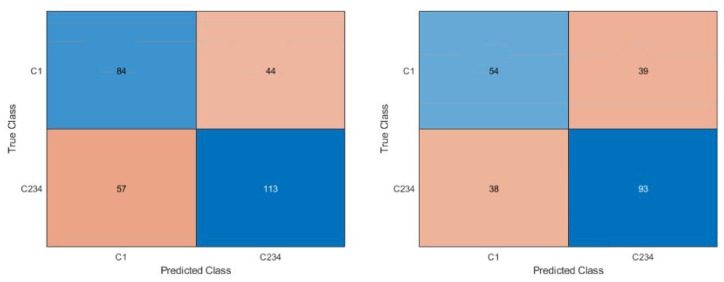
Confusion matrix for dataset 2, class 1 versus class 2, 3, 4. Right is all images, left is with glare removed. The blue diagonal shows correct classifications (predicted class matches the true class) while the orange off-diagonal shows misclassifications. Intensity of color is based off count in each category. Classes 2, 3, and 4 were combined to understand if the network was able to distinguish no corrosion versus any corrosion and understand the appropriate use case.

**Figure 6.**
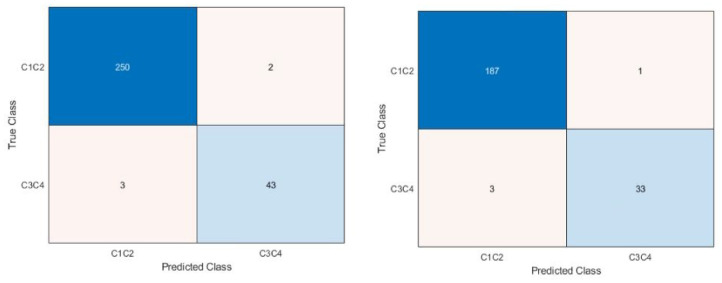
Confusion matrix for dataset 3, C12 versus C34. Right is all images, left is images with glare removed. The blue diagonal shows correct classifications (predicted class matches the true class) while the orange off-diagonal shows misclassifications. Intensity of color is based off count in each category. Classes 1 and 2 were combines, as were 3, and 4 were combined to understand if the network was able to distinguish between no/mild and moderate/severe corrosion and determine if class 2 was the main issue with distinguishing in the dataset 2 case. This determined the most appropriate use case to be as a screening tool to detect moderate/severe corrosion rapidly.

The confusion matrices are shown in Figures 4 to 6. Figure 4 shows the confusion matrices for the first dataset (class 1 vs 2 vs 3 vs 4). With or without the images with metallic glare excluded, the network failed to classify any test images as class 4. The most common misclassification was classifying as class 2 when the true image class was class 1. Class 1 never misclassified an image on the other end of the spectrum from it (i.e. class 4 images were never misclassified as class 1). Figure 5 shows the confusion matrices for second dataset (class 1 vs classes 2, 3, and 4). There were similar amounts of misclassifications for both classes.

Figure 6 shows the confusion matrices for the third dataset (classes 1 and 2 vs classes 3 and 4). This dataset had the fewest number of misclassifications, with little difference between if images with glare were included or not.

**Figure 7.**
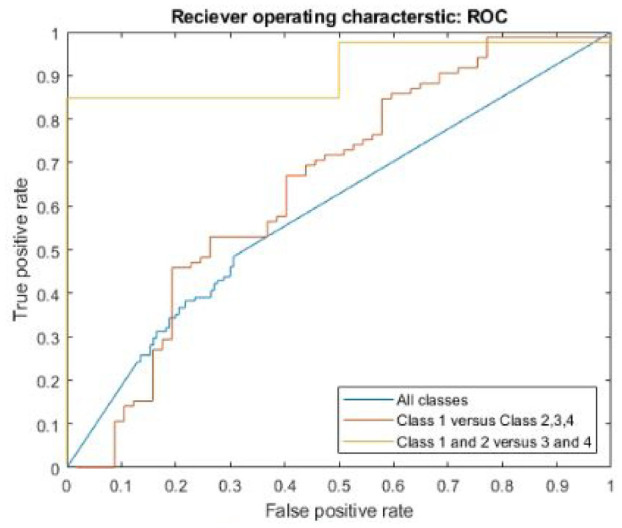
ROC for all datasets, with all images included.

The receiver operating characteristic was plotted and the plots for all datasets with all images are shown in Figure 7. The third dataset (classes 1 and 2 vs 3 and 4) had the greatest area under the curve and the fewest individual points, showing that the network made guesses with similar probabilities for many of the images. The second dataset (class 1 vs 2, 3 and 4) had a much lower area under the curve, with many more points, showing a range in the probabilities for different guesses. The first data set (class 1 vs 2 vs 3 vs 4) had the lowest area under the curve and showed a number or probabilities guessed, but significantly fewer outside the center of the graph.

The accuracy, sensitivity for each class, and area under the curve were computed for each dataset and for both versions of the testing set. Table 4 shows a summary of these metrics. Overall, the third dataset (class 1 and 2 vs class 3 and 4) had the greatest sensitivity, accuracy, and area under the curve for each class. The inability of the network to classify any images as class 4 for dataset one is reflected in the 0 sensitivity for class 4 in both versions of the testing set.

**Table 4.** Computed error metrics for each dataset.

Dataset	Sensitivity		Accuracy	Area under the curve (AUC)
1. Each corrosion class				
All images	C1	0.5667	48.21%	0.5941
	C2	0.4444		
	C3	0.3636		
	C4	0		
Glare removed	C1	0.5714	48.21%	0.6116
	C2	0.4500		
	C3	0.5333		
	C4	0		
2. No corrosion versus corrosion				
All images	C1	0.5957	66.11%	0.6875
	C234	0.7197		
Glare removed	C1	0.5869	65.63%	0.6661
	C234	0.7045		
No to mild corrosion versus moderate to severe corrosion				
All images	C12	0.9881	98.32%	0.9740
	C34	0.9556		
Glare removed	C12	0.9842	98.31%	0.9693
	C34	0.9706		

## Discussion

The ability to rapidly identify trunnions for further study is an important aspect in being able to achieve large-scale implant retrieval studies assessing taper corrosion with reduced labor. In this study, a convolutional neural network was developed that could discriminate corrosion that was absent or mild (Goldberg classes 1 and 2) from corrosion that was moderate or severe (Goldberg classes 3 and 4) with 98.32% accuracy.

The first dataset, which has the network classify the images into each Goldberg corrosion class separately, had the poorest performance. This dataset gives insight into the behavior of the network. Of note, the network failed to classify any images (correctly or incorrectly) as class 4 during testing. There were significantly fewer class 4 images available than any other class. This is unsurprising, as class 4 corrosion is the rare occurrence of severe corrosion that has >10% of taper surface containing black debris, pits, or etch marks.^5^ Although the network is trained with all available images, there was less opportunity for the network to learn from the class 4 images because they were so few in frequency. The images that were available for training may not have generalized well to the test set or there was so few that the network was never able to distinguish a high probability of class 4 for images. The confusion matrix also showed that there were several images that were classified as class 2 when their true class was class 1. The second dataset (class 1 vs classes 2, 3, and 4) showed a marginal improvement in its sensitivity for detecting class 1 images, but the area under the curve remained like the first dataset. When class 1 and class 2 images were combined in the third dataset (classes 1 and 2 vs 3 and 4), the network was able to perform with excellent accuracy, specificity, and a high area under the curve that showed excellent discrimination between the two classes.

The difficulty in discriminating between class 1 and class 2 Goldberg corrosion scoring could be attributed to the semi-quantitative nature of the Goldberg scale. Class 2 has the broadest definition of corrosion presence, with any discoloration up to 30% of the surface being considered. Small patches of discoloration or corrosion may be difficult to distinguish on the network and may point to segmentation being necessary to distinguish these. The results of the third dataset supports this, as when we combined class 1 and 2 versus 3 and 4, the network was able to distinguish with a high accuracy and very well. Segmentation is commonly used in other corrosion detection applications and in other applications before classification is done, but it was excluded here due to its intensity of requiring an observer to manually segment the training images.^31,32^

It was anticipated that the glare on images taken without the diffusion adapter would likely affect the network’s ability to discriminate the corrosion score. Despite this, there was little difference in the error metrics between having all the images and only images without glare for all cases. This is believed to be because the image scoring was done from the same photos as the network was given and the network was trained using images with glare as well. There was still a higher proportion of misclassifications when the glare images were included than when they were not. Best practice would be to take all images with a diffusion adapter, but it was removed in certain instances to ensure proper clearance between the stem and the lens. However, incorporation of less-than-perfect images improves the generalizability of the results. There were two main surface treatments observed: trunnions with machining lines and ones with a smooth surface. Both were included as they are both common in current implant designs, and the network performed well despite the surface difference due to the high volume of both surfaces in the collection. This was further shown when no trend seen amongst different trunnion designs and misclassification of images, thus the network generalizes well across the difference designs present in the data.

A previous attempt to automate damage scoring of trunnions was done by Milimonfared et al. with a reported accuracy of 85% and the ability to distinguish across the four classes using support vector machine learning.^24^ They imaged 138 stems, with a total of eight images per stem. A description of the image population in each class was not shared. Accuracy was reported but additional metrics such as sensitivity, confusion matrices, and area under the curve were not reported. Furthermore, it does not appear they ensured every class was present in their testing set. Although their method had high reported accuracy, without knowing information such as the sensitivity per class, it is difficult to determine if this pipeline could be used to reliably score implants. Kunze et al. have pointed out that underreporting of models and network evaluation is a common theme in machine learning studies in total joint arthroplasty and has called for more reliable reporting, including adequate reporting of results beyond accuracy.^33^ In contrast, our network can distinguish between no to mild corrosion versus moderate to severe corrosion with a higher accuracy than Milimonfared et al. , but it is unable to distinguish between the four classes effectively. We also evaluated 725 stems in our study in contrast to 138 in the Milimonfared et al. study. The increased reporting, including class population descriptions, confusion matrices, and class sensitivity characterize the network performance in our study to better understand the reliability of the network and where it may fail. This network can be reliably used as a screening tool to select implants for further study but in its current state cannot be used to classify across the full Goldberg scale.

Limitations of this study include an unbalanced dataset for testing and training. There was significantly more class 1 and 2 images available than class 3 and 4, and ideally a balanced dataset is best practice for neural network training. This network also does not represent a full automation of damage scoring across the Goldberg scores, which is sought after to further reduce labor barriers to large-scale studies and the need for skilled observers. The interclass correlation coefficient for interobserver reliability was considered only moderately reliable, but this is consistent with a previous study determining the reliability of scoring.^34^ The images trained were quite large (900 × 900 px), which limited the amount of convolutional layers to be included to control the trainable parameter number, which increases massively with each layer add and required more images to be able to sufficiently train. Further studies should strive to include more images to be able to train a larger network and achieve the task of being able to classify each class individually. The images that this network was trained and tested on were taken using a high-quality digital microscope. This microscope is unlikely to be found in a center that does not have a strong research focus and limits the accessibility of this network to be used at smaller centers. Further studies should look to incorporate images acquired using various acquisition systems, such as a smartphone, to improve accessibility of this network.

## Conclusions

In conclusion, a convolutional neural network was developed that could discriminate no and mild corrosion (Goldberg class 1 and 2) from moderate and severe corrosion (Goldberg class 3 and 4) with 98.32% accuracy, and a class 1 and 2 sensitivity of 0.9881 and class 3 and 4 sensitivity of 0.9556. The network, in its current form, was not successful in distinguishing each individual class. Its most suitable use is as a screening tool to discriminate class 1 and 2 implants from class 3 and 4, to help rapidly identify implants that should be considered for further study.

## Supplemental Material

sj-docx-1-pih-10.1177_09544119231177834 – Supplemental material for A convolutional neural network for high throughput screening of femoral stem taper corrosion Supplemental material, sj-docx-1-pih-10.1177_09544119231177834 for A convolutional neural network for high throughput screening of femoral stem taper corrosion by Anastasia M Codirenzi, Brent A Lanting and Matthew G Teeter in Proceedings of the Institution of Mechanical Engineers, Part H: Journal of Engineering in Medicine
