# Supplementary material for: A convolutional neural network for high throughput screening of femoral stem taper corrosion
Source: Proc Inst Mech Eng H. 2023 Jun 9;237(7):806–14. doi: 10.1177/09544119231177834 (PMC10350735; doi:10.1177/09544119231177834)
Supplement: sj-docx-1-pih-10.1177_09544119231177834 – Supplemental material for A convolutional neural network for high throughput screening of femoral stem taper corrosion [file sj-docx-1-pih-10.1177_09544119231177834.docx]

**Supplemental Information**

**Supplementary Tables**

| **Learning Rate Variance** | **Accuracy** | **Area Under the Curve** |
| --- | --- | --- |
| 0.001 | 88.93% | 0.7323 |
| **0.003** | **98.32%** | **0.9749** |
| 0.005 | 83.89% | 0.7327 |
| 0.008 | 85.23% | 0.7216 |
| 0.010 | 84.90% | 0.6000 |

Table S1: Variation of the learning rate for the presented network

| **Network Variations** | **Accuracy** | **Area Under the Curve** |
| --- | --- | --- |
| **Network as presented** | **98.32%** | **0.9749** |
| Without Batch Normalization | 87.25% | 0.5870 |
| Without L2 regularization | 82.21% | 0.7377 |
| Without Early Stopping | 78.52% | 0.6125 |
| Stochastic Gradient Descent | 84.56% | 0.5000 |

Table S2: Variation of the network architecture

**Supplementary Discussion**

From Tables S1 and S2 we can see that the network is sensitive to both parameter and hyperparameter changes. These tables are representative of the trial and error process conducted in order to arrive to the network presented in the main text and demonstrated using the dataset which combined class 1/class 2 and class 3/class 4.
